# Natural course of scoliosis in proximal spinal muscular atrophy type II and IIIa: descriptive clinical study with retrospective data collection of 126 patients

**DOI:** 10.1186/1471-2474-14-283

**Published:** 2013-10-04

**Authors:** Albert Fujak, Wolfgang Raab, Alexander Schuh, Silvia Richter, Raimund Forst, Jürgen Forst

**Affiliations:** 1 Department of Orthopaedic Surgery, Friedrich-Alexander-Universität Erlangen-Nürnberg, Rathsberger Str. 57, D-91054 Erlangen, Germany; 2 Medizinische Klinik II-4, Klinikum Nürnberg, Breslauer Str. 201, Nuremberg D-90471, Germany; 3 Neumarkt Hospital, Teaching Hospital of the Friedrich-Alexander-Universität Erlangen-Nürnberg, Str. 12, D-92318 Neumarkt i.d. OPf, Erlangen, Germany

**Keywords:** Spinal muscular atrophy, Scoliosis, Pelvic obliquity, Pulmonary function, Natural course

## Abstract

**Background:**

Progressive scoliosis, pelvic obliquity and increasing reduction of pulmonary function are among the most significant problems for patients with SMA type II and SMA type III once they have lost the ability to walk. The aim of this study was to examine and document the development and natural course of scoliosis in patients with spinal muscular atrophy type II and IIIa.

**Methods:**

For the purposes of a descriptive clinical study, we observed 126 patients, 99 with SMA II and 27 with SMA IIIa and the data of scoliosis, pelvic obliquity and relative age-dependent inspiratory vital capacity were evaluated.

**Results:**

Scoliosis and pelvic obliquity were regularly observed already in children under 4 years old in the group with SMA II. The severity and progression of both conditions were much more pronounced in the SMA II group than in the IIIa group. There was already a distinct reduction in relative vital capacity in the group of 4- to 6-year-olds with SMA II.

**Conclusions:**

The differences between the two SMA types II and IIIa described in this study should be taken into consideration when developing new treatments and in management of scoliosis in the childhood years of these patients.

## Background

Proximal spinal muscular atrophy (SMA) is a hereditary autosomal recessive disease with muscular hypotonia, hyporeflexia, symmetrical weakness and atrophy of the skeletal muscles due to chronically degenerative alterations in the anterior horn cells. It is caused by a deletion or mutation in the telomeric survival motor neuron gene (telSMN-gene, SMN1) on chromosome 5q [1,2].

Table 1 shows the classification of proximal SMA we use in our study. Children with type II are unable to walk but they are or were able to sit unsupported. In some cases they are able to stand with the support of assistive devices. The onset of SMA II usually occurs before 18 months of age. Children with type IIIa and IIIb are or were able to walk. Type IIIa manifests itself up to the age of three years and type IIIb over the age of three years to the age of thirty years. The subdivision of type III is in our opinion important for children’s orthopaedics. The course of the disease and treatment strategies are different in types IIIa and IIIb [3].

**Table 1:** **Classification and prognosis of different types of autosomal recessive proximal spinal muscular atrophy**[1,2]

**Types**		**Manifestation**	**Functional grading**	**Life expectancy**
Ia	more severe	prenatal (30%) to 3-6 months	unable to roll over or sit	< 30 months = 100%
				< 18 months = 95%
				< 7 months = 50%
Ib	less severe	like Ia	like Ia	2.5 - 20 years *
II	intermediate	birth -18 months	sitting	2.5 - 30 years *
IIIa	mild, retarded motor development	to 3 years	walking	4th – 6th decade, usually normal
IIIb	mild, normal motor development	> 3 - 30 years	walking	
IV	adult	> 30 years	walking	normal

* Longer life expectancy is quite possible.

The fundamental orthopaedic problem for patients with SMA type II and IIIa is progressive scoliosis with increasing pelvic obliquity and seated instability, reduced lung function and contractures in the lower and upper extremities [4-8].

To date there is no causal therapy for SMA. The course of the disease and the patient’s quality of life can be greatly improved by established medical procedures [3,5,6]. Orthopaedic treatment of scoliosis and pelvic obliquity is of great importance for SMA patients. It includes conservative methods such as corsets up to the point when surgery is definitely indicated, in order to improve stability temporarily when seated, and surgical stabilisation of the spine.

The purpose of this study is to determine the development and course of scoliosis, pelvic obliquity and vital capacity in patients with the intermediate SMA form (type II) and mild SMA form with retarded motor development (type IIIa) following a survey of 126 patients. It is becoming increasingly difficult to find such data since spinal surgery is nowadays being carried out on many children at ever earlier ages.

## Methods

The data were collected during special nationwide orthopaedic consultations for patients with neuromuscular disorders from 1999 to 2010. All SMA patients with evident scoliosis without surgery examined during that time period were included in this study. 126 patients with SMA, 99 with SMA type II and 27 with SMA type IIIa, 61 male patients and 65 female were evaluated. 41 patients, 37 with SMA type II and 4 with type IIIa, were excluded because they had already had spine surgery.

Diagnosis was confirmed either genetically (79 patients) or by muscle biopsy and neurophysiology (47 patients). The diagnosis in the older patients was confirmed by muscle biopsy and neurophysiology because genetic diagnostics in SMA developed gradually after 1995.

The average age at diagnosis was 1.6 years (± 0.9, 0.1 – 6) in the SMA II group and 3.1 years (± 1.5, 1.5 – 6.9) in the SMA IIIa group. For this study we examined children and young people from infancy to 20 years old.

The SMA II patients were by definition never able to walk unaided. We found that 24 SMA II patients started to sit unsupported at an average age of one year (± 1.3, 0.3 – 6, median 0.6). Many patients were able to sit unsupported by their last follow-up examination.

20 patients with SMA IIIa began to walk unaided at 1.4 years on average (± 0.6, 0.8 – 3, median 1.3) and 15 patients from this group were unable to walk unaided at an average age of 7.4 years (± 3.9, 1.5 – 14, median 8).

The scoliosis and pelvic obliquity were given clinical and radiological examinations. The radiographs were taken in two planes in a sitting position. A spirometry test was carried out from the age of 4 to 6 in order to assess pulmonary function. In this article, the values of the relative age-dependent inspiratory vital capacity (VC IN%) are evaluated.

This is a descriptive clinical study with retrospective data collection.

This study is in accordance with the ethical standards of the relevant local Ethics Commission (Research Ethics Committee, Faculty of Medicine, Department of Orthopaedic Surgery, Friedrich-Alexander-Universität Erlangen-Nürnberg, EK_No. 61_12 Bc).

## Results

Of the 99 patients with SMA II, 87 had C-shaped scoliosis (84 major thoracolumbar curves, 2 thoracic and one lumbar) and 12 had S-shaped scoliosis. Of the 27 patients with SMA IIIa, 18 had C-shaped scoliosis (all major thoracolumbar curves) and 9 (33%) had S-shaped scoliosis.

Of the S-shaped scoliosis, the main curve in SMA II patients was thoracic in 6 cases, lumbar in 4 and thoracolumbar in 2. In type IIIa patients, the main curve was thoracic in 6 cases and lumbar in 3.

Of the C-shaped scoliosis, there were 56 curves to the left and 31 right convex in type II patients, and 10 curves to the right and 8 left convex in type IIIa patients. The main curve of S-shaped scoliosis in type II was right convex in 4 cases and left convex in 8 cases. The main curve in 6 type IIIa patients with S-shaped scoliosis was right convex and left convex in 3 patients.

We regularly observed scoliosis and pelvic obliquity in the group with SMA II, even in children in the age group 0 to 4 years old. The maximum curve in this young group was 40° and the pelvic tilt was 10° (Table 2). The average scoliosis of the 14- to 15-year-olds was 82° with a maximum of 112°. The pelvic tilt in this age-group reached an average of 26° and a maximum of 45° (Table 2).

**Table 2:** Average main curvature and pelvic obliquity in SMA II and IIIa non-surgery patients

**Patient’s age (years)**	**n**	**Ø, ±, min – max, median**	**n**	**Ø, ±, min – max, median**
Main curve of the scoliosis (Cobb angle °)				
	SMA II (N = 58)		SMA IIIa (N = 12)	
0 - 4	9	Ø 26, ± 7, 15 - 40, 25	1	28
5 - 6	10	Ø 42, ± 19, 12 - 70, 37	2	28 and 32
7 - 10	25	Ø 63, ± 32, 17 - 138, 58	3	Ø 34, ± 8.5, 25 - 42, 35
11 - 13	6	Ø 74, ± 16, 56 - 102, 72.5	2	42 and 51
14 - 15	4	Ø 82, ± 28, 50 - 112, 83.5	1	54
16 - 20	4	Ø 73, ± 18, 58 - 100, 67.5	3	Ø 45, ± 8, 40 - 54, 42
Pelvic obliquity (°)				
	SMA II (N = 72)		SMA IIIa (N = 18)	
0 - 4	5	Ø 7, ± 4.5, 0 - 10, 10	-	-
5 - 6	12	Ø 17, ± 7, 0 - 30, 15	1	10
7 - 10	36	Ø 18, ± 10, 0 - 45, 15	3	Ø 10, ± 10, 0 - 20, 10
11 - 13	7	Ø 19, ± 15, 0 - 45, 20	5	Ø 10, ± 10, 0 - 20, 10
14 - 15	8	Ø 26, ± 13, 0 - 45, 27.5	5	Ø 8, ± 11, 0 - 20, 0
16 - 20	4	Ø 24, ± 18, 0 - 40, 27.5	4	Ø 14, ± 5, 10 - 20, 12.5

The prevalence, severity and progression of the scoliosis and pelvic obliquity were much more marked in SMA II patients than in the SMA IIIa group (Table 2).

Likewise, there was already a distinct reduction to an average of 54% in the relative vital capacity among the 4- to 6-year-olds with SMA II (Table 3). In comparison, we observed considerably better values in the SMA IIIa group, even among the older children (Table 3). The reduction of vital capacity was progressive with age in both groups but distinctly much more pronounced among SMA II patients than SMA IIIa (Table 3).

**Table 3:** Relative age-dependent inspiratory vital capacity (VC IN%) in SMA II and IIIa non-surgery patients

**VC IN%**	**SMA II (N = 40)**		**SMA IIIa (N = 15)**	
Patient’s age (years)	n	Ø, ±, min – max, median	n	Ø, ±, min – max, median
4 - 6	5	Ø 54, ± 13, 41 - 70, 58	-	-
7 - 10	19	Ø 44, ± 21, 14 - 91, 43	4	Ø 80, ± 23, 56 - 108, 78
11 - 13	9	Ø 37, ± 22, 10 - 86, 32	5	Ø 56, ± 24, 17 - 73, 70
14 - 15	3	Ø 37, ± 11, 25 - 47, 39	6	Ø 43, ± 11, 31 - 63, 40
16 - 20	4	Ø 42, ± 18, 25 - 63, 43	-	-

## Discussion

This study documents that scoliosis in patients with SMA II and SMA IIIa develops early and progresses rapidly and that their vital capacity deteriorates during childhood. The difference in how the conditions progress in the two groups becomes clear. In general, we verified that the course of scoliosis, pelvic obliquity and pulmonary function is on average milder in SMA IIIa patients than in SMA II patients [9,10]. C-shaped scoliosis predominates in both groups but there is a larger proportion of S-shaped curves among the IIIa group (33%) than among the SMA II group (12%).

Since surgical treatment for SMA patients who are unable to walk and who have progressive scoliosis with pelvic tilt is now well-established from the age of about 10 to 12 years old, there is less and less data about the natural course of the condition at our disposal [2,4,5,11-22]. However, we know from earlier studies that scoliosis increases over the course of time, leading to the loss of stability when sitting and a negative impact on everyday activities [9-13,15,17,18].

Our observations correlate with earlier studies [9,10,17,19,20]. Granata et al. [9] state that scoliosis in SMA II patients progresses by 8° per year and in SMA IIIa patients unable to walk by 3° per year [9,17]. According to Rodillo et al. [10], scoliosis in SMA patients progresses by between 5° and 15° per year.

Fitting a corset cannot halt the progression of scoliosis in SMA [10-18,23]. It can only improve seated stability temporarily, replace the use of arms to support the seated position and help to bridge the time until surgical stabilisation of the spine.

Our group of patients is made up of patients of different ages who came to our nationwide special orthopaedic consultations for neuromuscular disorders. Some of these patients have been regularly in our orthopaedic care since early childhood. This is a biased cohort of patients because our patients had visited us because of orthopaedic problems like contractures and scoliosis. The disadvantage of this study is the small amount of data, especially for small children and for older adolescents. Many children were given only a clinical examination, despite their existing scoliosis. X-ray images were not taken at every examination since they would have had no therapeutic effect. X-ray examinations were mostly made for initial findings, before a corset was supplied or where an indication for surgical stabilisation of the spine was in question.

Testing vital capacity in small children was not always possible, sometimes because they were unwilling to comply and sometimes because of technical reasons: there was often no mouthpiece for the spirometer to suit the children’s size and anatomy.

41 patients had already had surgical stabilisation of the spine at an average age of 9.9 years (4.8 -22.7) [24]. Data about older adolescents with severe scoliosis could be gathered only if the patients presented at our clinic for the first time at a later age or if earlier surgical treatment had been declined or had not been possible. It is for these reasons that data about patients older than 13 years is less conclusive. The course of the data for this age-group in Tables 2 and 3 is largely caused by the fact that most patients with severe scoliosis had already been operated on.

This study has made clear that the vital capacity of both SMA II and SMA IIIa patients decreases systematically as they grow [10,13,19,20]. However, there are already 4- to 6-year-olds with SMA II with marked reduction which we mostly see in the SMA IIIa group after 10 years old. The pulmonary function values in young people with SMA II seem to stabilise after their greatest time of growth. We have seen some patients who have kept stable values far into adult life.

This study presents cross sectional data, so that it cannot be concluded that every SMA patient with SMA type II or IIIa would experience exactly the same course as described. Significant individual differences are observed.

The mean age of sitting independently at an average age of one year seems to be surprisingly late for SMA II patients. It could be an indication for a biased cohort of patients, referred to the evaluated group due to the fact that scoliosis had already developed. Delayed sitting may be predictive of the severity of weakness indicating a higher risk for scoliosis.

This study highlights the need for a comprehensive strategy to prospectively identify and follow all SMA patients from an early age and monitor them for the course of disease, development and progression of scoliosis, pelvic obliquity and the respiratory insufficiency. We recommend starting to monitor of SMA patients regularly by clinical examination in the special orthopaedic consultation as early as possible to determine an individual strategy in the provision of corsets, orthoses and orthopaedic technical devices and to determine the time for operative spine stabilisation [3]. The aims of the orthopaedic management are stabilise, optimise and maintain the sitting and standing position and the ability to walk [3]. If scoliosis will be diagnosed in the clinical examination the radiographs in two planes in a sitting position without corset should be done to verify the scoliosis [24]. The course of scoliosis and pelvic obliquity could be monitored and documented by clinical examination. We recommend monitoring the patients every 3 to 12 months depending on the dynamics of the disease and child development and growth. Usually radiographs are not necessary with each examination. We recommend radiographs for special circumstances, such as the provision of corsets or an indication for spinal surgery [3,24].

The results of this study can be used as the basis for the evaluation and further development of surgical treatments of scoliosis in SMA patients, particularly in their early growth period [24-26]. Here it is important to know the differences between the two SMA types, type II and IIIa, in order to calculate the expected course of the condition, and to choose the best treatment and the right time for surgery.

## Conclusions

Differentiating SMA types II, IIIa and IIIb is very important in order to be able to assess correctly the prognosis of the development of scoliosis, pelvic obliquity and pulmonary insufficiency. The development of scoliosis, pelvic obliquity and reduction of pulmonary function in patients with SMA II and IIIa are investigated and compared accurately. Patients with SMA II are clearly affected earlier and more distinctly than patients with SMA IIIa, making the treatment of SMA II more difficult.

## Competing interests

The authors declare that they have no competing interests.

## Authors’ contributions

AF, JF, RF and SR examined the patients and collected the data. WR assisted in examination of patients, collected and prepared the data. JF and RF were the clinical and academic supervisors. AF and WR wrote the first draft. All authors were involved in the interpretation of data, preparation and revision of manuscript. All authors read and approved the final manuscript.

## Pre-publication history

The pre-publication history for this paper can be accessed here:

http://www.biomedcentral.com/1471-2474/14/283/prepub
